# Supplementary material for: ChatGPT as a Tool for Biostatisticians: A Tutorial on Applications, Opportunities, and Limitations
Source: Stat Med. 2025 Oct 23;44(23-24):e70263. doi: 10.1002/sim.70263 (PMC12548020; doi:10.1002/sim.70263)
Supplement: Supplementary file 1 — Data S1. Supporting Information. [file SIM-44-0-s001.zip › Supplementary Materials Additional.zip!/3.2_Diagnostic_Test/3.2_Diagnostic_Chat7.pdf]

I would like to determine sensitivity and specificity of two diagnostic tests. I do have the diagnostic test outcomes, but I do not know the gold standard. What can I do?

Please, tell me more about the latent class analysis.

I have two populations with two different disease prevalences. I have two diagnostic tests which are conditional independent. I would like to get the log-likelihood of this setting.

What is the Hui-Walter paradigm?

You've posed a very good and important set of questions — let's go through them step by step.

❓ Your problem:

You have:
✅ two diagnostic tests (outcomes known)
✅ two populations (with different prevalences)
⛔ *no* gold standard (the true disease status is unknown)

You want to:

1. Estimate sensitivity and specificity of the tests
2. Understand latent class analysis (LCA)
3. Obtain the log-likelihood of your setting
4. Understand the Hui-Walter paradigm

📌 Why is this a problem?

Without a gold standard, it's not directly possible to compute sensitivity/specificity the usual way (which is comparing test result to true disease state).

📌 Solution approach: Latent Class Analysis (LCA)

🚩 1️⃣ What is Latent Class Analysis (LCA)?

Latent Class Analysis is a statistical model that assumes the existence of an *unobserved* (latent) variable — in this case, the true disease status — that explains the observed test results.

In this context:

- The *latent class* = whether a person is diseased or not (unobserved)
- The *observed variables* = results of the two tests

You model the joint distribution of the observed tests **as a function of**:

- The disease prevalence in each population
- The sensitivity and specificity of each test

If you can model this joint distribution and fit it to your data, you can then estimate sensitivities, specificities, and prevalences **without knowing the true disease status**.

🚩 2️⃣ The Hui-Walter paradigm

This is the *classic framework* for LCA with diagnostic tests without a gold standard, proposed by Hui & Walter (1980).

Assumptions of Hui-Walter model:

1. Two or more populations (with different prevalences)
2. Two or more diagnostic tests
3. Conditional independence of the tests, given disease status
4. The sensitivities and specificities of the tests are the *same* across populations

Under these assumptions, **the model is identifiable** — meaning, you can uniquely estimate sensitivity, specificity, and prevalence.

🚩 3️⃣ Model structure and log-likelihood

✏️ Model ingredients:

Notation:

- Populations: $k = 1, 2$
- Tests: $T_1, T_2 \rightarrow$ outcomes: 1 (positive), 0 (negative)
- Latent disease status: $D = 1$ (diseased), $D = 0$ (non-diseased)

Parameters:

- $\pi_k = P(D = 1 \text{ in population } k)$
- Se_1, Se_2 = sensitivities of tests 1 and 2
- Sp_1, Sp_2 = specificities of tests 1 and 2

✏️ Conditional independence:

For a subject in population k, the probability of observing any pattern of test results is:

$$P(T_1 = t_1, T_2 = t_2 | \text{population } k) = \pi_k \cdot P(T_1 = t_1 | D = 1) P(T_2 = t_2 | D = 1) + (1 - \pi_k) \cdot P(T_1 =$$

Where:

$$P(T_j = 1 | D = 1) = Se_j, \quad P(T_j = 0 | D = 1) = 1 - Se_j$$

$$P(T_j = 1|D = 0) = 1 - Sp_j, \quad P(T_j = 0|D = 0) = Sp_j$$

✏️ Likelihood and log-likelihood

Let $n_{k,ij}$ be the number of subjects in population k with test outcomes:

- i = 1 if T_1 positive, 0 otherwise
- j = 1 if T_2 positive, 0 otherwise

You can then write the likelihood as:

$$L = \prod_{k=1}^{2} \prod_{i=0}^{1} \prod_{j=0}^{1} (P(T_1 = i, T_2 = j|\text{population } k))^{n_{k,ij}}$$

And the **log-likelihood** is:

$$\log L = \sum_{k=1}^{2} \sum_{i=0}^{1} \sum_{j=0}^{1} n_{k,ij} \cdot \log (P(T_1 = i, T_2 = j|\text{population } k))$$

🚩 4️⃣ Identifiability

In this setting, you have:

- 2 populations
- 2 tests → 4 observable response patterns per population
- $2 \times 4 = 8$ observable frequencies

Parameters:

- Se_1, Se_2 → 2
- Sp_1, Sp_2 → 2
- π_1, π_2 → 2
  → Total 6 parameters

Since you have 8 independent pieces of information (after subtracting constraints like sum-to-total), the model is **identifiable** under Hui-Walter assumptions.

Summary:

✅ You can use **Latent Class Analysis** to estimate the parameters
✅ The **Hui-Walter paradigm** provides a framework to make the model identifiable
✅ You can write the **log-likelihood** explicitly and fit the model (e.g., by maximum likelihood or Bayesian methods)

Final comment:

There are R packages that help with this, e.g.:

- `poLCA`
- `randomLCA`
- `LCMCR` (for Bayesian LCA)
- Or you can code the log-likelihood manually and optimize it

---

Would you like me to:

1. Write out a concrete example with numbers?
2. Show R code to fit this model?
3. Show how the identifiability works step-by-step?
4. Give references for further reading?

Just tell me how deep you want to go. 🚀

> I would like to see a worked example and a R script that implements this model.
>
> I would like to add confidence intervals to the estimated sensitivities, specificities, and prevalences.
>
> I would like to run a bootstrap version for robust confidence intervals.

Excellent — this is a very good plan!

Let's go step-by-step:
👉 I'll give you:
1️⃣ A *worked example* with artificial data
2️⃣ An R script that:

- Fits the Hui-Walter model by *maximum likelihood*
- Estimates sensitivities, specificities, and prevalences
- Adds *confidence intervals* using:
  - **Fisher information (Wald intervals)**
  - **Bootstrap intervals** (robust)

🌟 1️⃣ Worked Example

✏️ Setup

We have:

- **2 populations** (Pop1 and Pop2), different prevalences
- **2 tests** → binary outcomes: (T1, T2)

True parameters (unknown to model — we simulate them!):

Parameter	Value
Sensitivity Test 1 Se_1	0.85
Specificity Test 1 Sp_1	0.90
Sensitivity Test 2 Se_2	0.80
Specificity Test 2 Sp_2	0.95
Prevalence Pop 1 π_1	0.3
Prevalence Pop 2 π_2	0.6

✏️ Data generation

For each population, for each subject:

1. Simulate disease status $D \sim \text{Bernoulli}(\pi_k)$
2. Simulate T1, T2 conditional on D, using Se and Sp

Finally, we count **joint test results**:

- $(T1, T2) = (0,0), (0,1), (1,0), (1,1)$

🌟 2️⃣ R Script

Now here is a **full R script** that does everything you asked for:

```r
# Load packages
library(tidyverse)
library(boot)

# Simulate data
set.seed(123)

# True parameters
Se1 <- 0.85
Sp1 <- 0.90
Se2 <- 0.80
Sp2 <- 0.95
pi1 <- 0.3
pi2 <- 0.6

# Sample sizes
n1 <- 1000
n2 <- 1000

# Function to simulate one population
simulate_population <- function(n, pi, Se1, Sp1, Se2, Sp2) {
  D <- rbinom(n, 1, pi)
  T1 <- ifelse(D == 1, rbinom(n, 1, Se1), rbinom(n, 1, 1 - Sp1))
  T2 <- ifelse(D == 1, rbinom(n, 1, Se2), rbinom(n, 1, 1 - Sp2))
  data.frame(D, T1, T2)
}

# Simulate populations
pop1 <- simulate_population(n1, pi1, Se1, Sp1, Se2, Sp2) %>% mutate(pop = 1)
pop2 <- simulate_population(n2, pi2, Se1, Sp1, Se2, Sp2) %>% mutate(pop = 2)
```

```
# Combine
data <- bind_rows(pop1, pop2)

# Create contingency tables
table_data <- data %>%
  group_by(pop, T1, T2) %>%
  summarise(count = n()) %>%
  ungroup()

print(table_data)

# Likelihood function
loglik <- function(params, table_data) {
  # Parameters to estimate
  Se1 <- params[1]
  Sp1 <- params[2]
  Se2 <- params[3]
  Sp2 <- params[4]
  pi1 <- params[5]
  pi2 <- params[6]

  logL <- 0

  for (k in 1:2) {
    pi_k <- ifelse(k == 1, pi1, pi2)

    sub_table <- table_data %>% filter(pop == k)

    for (i in 0:1) {
      for (j in 0:1) {
        count_ij <- sub_table %>% filter(T1 == i, T2 == j) %>% pull(count)
        if (length(count_ij) == 0) count_ij <- 0

        # Probability of (i,j) pattern
        p_ij <- pi_k *
          (ifelse(i == 1, Se1, 1 - Se1)) *
          (ifelse(j == 1, Se2, 1 - Se2)) +
          (1 - pi_k) *
          (ifelse(i == 1, 1 - Sp1, Sp1)) *
          (ifelse(j == 1, 1 - Sp2, Sp2))

        # Avoid log(0)
        p_ij <- max(p_ij, 1e-10)

        # Add to log likelihood
        logL <- logL + count_ij * log(p_ij)
      }
    }
  }

  return(-logL)  # Negative log-likelihood for minimization
}

# Initial values
init_params <- c(0.8, 0.8, 0.8, 0.8, 0.5, 0.5)

# Fit model
fit <- optim(par = init_params,
             fn = loglik,
             table_data = table_data,
             method = "L-BFGS-B",
             lower = rep(0.01, 6),
             upper = rep(0.99, 6),
             hessian = TRUE)

# Estimated parameters
est_params <- fit$par
names(est_params) <- c("Se1", "Sp1", "Se2", "Sp2", "pi1", "pi2")

# Print estimates
print(est_params)

# Wald confidence intervals (based on Hessian)
se_params <- sqrt(diag(solve(fit$hessian)))
```

```
wald_ci <- tibble(
  parameter = names(est_params),
  estimate = est_params,
  lower = est_params - 1.96 * se_params,
  upper = est_params + 1.96 * se_params
)

print(wald_ci)

# --- Bootstrap for robust CI ---

# Bootstrap function
bootstrap_fn <- function(data, indices) {
  data_boot <- data[indices, ]

  table_boot <- data_boot %>%
    group_by(pop, T1, T2) %>%
    summarise(count = n()) %>%
    ungroup()

  fit_boot <- optim(par = init_params,
                    fn = loglik,
                    table_data = table_boot,
                    method = "L-BFGS-B",
                    lower = rep(0.01, 6),
                    upper = rep(0.99, 6))

  return(fit_boot$par)
}

# Prepare data in long form for bootstrap
data_long <- data

# Run bootstrap
set.seed(123)
boot_res <- boot(data = data_long, statistic = bootstrap_fn, R = 500)

# Bootstrap CIs (percentile)
boot_ci <- tibble(
  parameter = names(est_params),
  estimate = est_params,
  lower = apply(boot_res$t, 2, quantile, probs = 0.025),
  upper = apply(boot_res$t, 2, quantile, probs = 0.975)
)

print(boot_ci)
```

🌟 3️⃣ Summary

This script does:
✅ Simulate 2 populations with 2 tests
✅ Tabulate the joint test outcomes
✅ Define and fit the **Hui-Walter model**
✅ Obtain *maximum likelihood estimates* of:

- Sensitivity
- Specificity
- Prevalence

✅ Calculate:

- **Wald CIs** (based on Fisher information / Hessian)

- **Bootstrap CIs** (robust)

Final notes:

1️⃣ This is a *basic Hui-Walter model* — you can extend it (for example with **Bayesian LCA** — more flexible).
2️⃣ You can increase the **number of populations** or **number of tests**.
3️⃣ If tests are not conditionally independent → more complex models (latent class with local dependence).

Would you like me to also provide:
✅ A version using **Bayesian LCA** (with Stan or JAGS)?
✅ A version with **3 tests**?
✅ A **more user-friendly R function** you can reuse?

Just tell me! 🚀

I would like to see a worked example and a R script that implements this model.

I would like to add confidence intervals to the estimated sensitivities, specificities, and prevalences.

I would like to run a bootstrap version for robust confidence intervals.

Perfect — your request is **very clear**. I will now give you a *full worked example*, exactly as you asked:

🚩 Overview

You want an R script that does:
✅ Simulate data under the Hui-Walter paradigm
✅ Fit the latent class model (2 tests, 2 populations, no gold standard)
✅ Estimate sensitivity, specificity, and prevalence
✅ Provide **confidence intervals**:

- via Fisher information (Wald)
- via **bootstrap** (robust)

🚩 Full Worked Example

⚙️ Step 1 — Simulate data

We assume:

- 2 populations (different prevalences)
- 2 diagnostic tests (conditionally independent)

📜 R SCRIPT

```r
r

# Load necessary packages
library(tidyverse)
library(boot)

# ---------------------------------------------
# STEP 1: Simulate data under Hui-Walter model
# ---------------------------------------------

# Set true parameters
true_params <- list(
  Se1 = 0.85,
  Sp1 = 0.90,
  Se2 = 0.80,
  Sp2 = 0.95,
  pi1 = 0.30,
  pi2 = 0.60
)

# Simulate one population
simulate_population <- function(n, pi, Se1, Sp1, Se2, Sp2) {
  D <- rbinom(n, 1, pi)
  T1 <- ifelse(D == 1, rbinom(n, 1, Se1), rbinom(n, 1, 1 - Sp1))
  T2 <- ifelse(D == 1, rbinom(n, 1, Se2), rbinom(n, 1, 1 - Sp2))
  data.frame(D, T1, T2)
}

# Simulate data
set.seed(123)

n1 <- 1000
n2 <- 1000

pop1 <- simulate_population(n1, true_params$pi1, true_params$Se1, true_params$Sp1,
true_params$Se2, true_params$Sp2) %>%
  mutate(pop = 1)
pop2 <- simulate_population(n2, true_params$pi2, true_params$Se1, true_params$Sp1,
true_params$Se2, true_params$Sp2) %>%
  mutate(pop = 2)

# Combine data
data_all <- bind_rows(pop1, pop2)

# Create contingency table
table_data <- data_all %>%
  group_by(pop, T1, T2) %>%
  summarise(count = n()) %>%
  ungroup()

print(table_data)

# ---------------------------------------------
# STEP 2: Define log-likelihood for Hui-Walter model
# ---------------------------------------------

loglik <- function(params, table_data) {
  Se1 <- params[1]
  Sp1 <- params[2]
  Se2 <- params[3]
  Sp2 <- params[4]
  pi1 <- params[5]
  pi2 <- params[6]

  logL <- 0

  for (k in 1:2) {
    pi_k <- ifelse(k == 1, pi1, pi2)

    sub_table <- table_data %>% filter(pop == k)
```

```
    for (i in 0:1) {
      for (j in 0:1) {
        count_ij <- sub_table %>% filter(T1 == i, T2 == j) %>% pull(count)
        if (length(count_ij) == 0) count_ij <- 0

        # Probability of (i,j) pattern
        p_ij <- pi_k *
          (ifelse(i == 1, Se1, 1 - Se1)) *
          (ifelse(j == 1, Se2, 1 - Se2)) +
          (1 - pi_k) *
          (ifelse(i == 1, 1 - Sp1, Sp1)) *
          (ifelse(j == 1, 1 - Sp2, Sp2))

        p_ij <- max(p_ij, 1e-10)  # Avoid log(0)

        logL <- logL + count_ij * log(p_ij)
      }
    }
  }

  return(-logL)  # Negative log-likelihood
}

# ---------------------------------------------
# STEP 3: Fit model (maximum likelihood)
# ---------------------------------------------

# Initial values
init_params <- c(0.8, 0.8, 0.8, 0.8, 0.5, 0.5)

# Optimization
fit <- optim(par = init_params,
             fn = loglik,
             table_data = table_data,
             method = "L-BFGS-B",
             lower = rep(0.01, 6),
             upper = rep(0.99, 6),
             hessian = TRUE)

# Estimated parameters
est_params <- fit$par
names(est_params) <- c("Se1", "Sp1", "Se2", "Sp2", "pi1", "pi2")

# Print estimates
cat("\n--- Parameter estimates ---\n")
print(est_params)

# ---------------------------------------------
# STEP 4: Wald confidence intervals
# ---------------------------------------------

# Standard errors from Hessian
se_params <- sqrt(diag(solve(fit$hessian)))

# Wald CIs
wald_ci <- tibble(
  parameter = names(est_params),
  estimate = est_params,
  lower = est_params - 1.96 * se_params,
  upper = est_params + 1.96 * se_params
)

cat("\n--- Wald confidence intervals ---\n")
print(wald_ci)

# ---------------------------------------------
# STEP 5: Bootstrap for robust CIs
# ---------------------------------------------

# Bootstrap function
bootstrap_fn <- function(data, indices) {
  data_boot <- data[indices, ]

  table_boot <- data_boot %>%
    group_by(pop, T1, T2) %>%
```

```
    summarise(count = n()) %>%
    ungroup()

  fit_boot <- optim(par = init_params,
                    fn = loglik,
                    table_data = table_boot,
                    method = "L-BFGS-B",
                    lower = rep(0.01, 6),
                    upper = rep(0.99, 6))

  return(fit_boot$par)
}

# Prepare data in long form
data_long <- data_all

# Run bootstrap
set.seed(123)
boot_res <- boot(data = data_long, statistic = bootstrap_fn, R = 500)

# Bootstrap percentile CIs
boot_ci <- tibble(
  parameter = names(est_params),
  estimate = est_params,
  lower = apply(boot_res$t, 2, quantile, probs = 0.025),
  upper = apply(boot_res$t, 2, quantile, probs = 0.975)
)

cat("\n--- Bootstrap confidence intervals ---\n")
print(boot_ci)
```

🚩 Summary of script

✅ Simulates data for 2 populations and 2 tests
✅ Fits **Hui-Walter latent class model** (maximum likelihood)
✅ Gives:

- Point estimates of Se, Sp, pi
- Wald CIs (based on Fisher information)
- **Bootstrap CIs** (robust!)

🚩 How to run

- Copy full script to R
- Run the whole script → you will see:
  - Estimated Se1, Sp1, Se2, Sp2, pi1, pi2
  - Wald CIs
  - Bootstrap CIs

🚩 Extensions

If you want, I can also give you:
✅ Version with **3 tests**
✅ **Bayesian version** (with Stan or JAGS) → more flexible, handles dependence

✅ Version with **more than 2 populations**
✅ Code as a **clean R function** you can call easily

Would you like me to generate one of those next? Just tell me what direction you want — I am happy to build it for you. 🚀

Please provide a comprehensive R code for the issues discussed in this session.

Absolutely! Below is a **comprehensive, fully-documented R script** that puts everything together from our session:

🚀 Hui-Walter Model: Full R Code

Goals:
✅ Simulate 2 populations
✅ 2 diagnostic tests (conditionally independent)
✅ No gold standard
✅ Fit Hui-Walter latent class model
✅ Estimate sensitivity, specificity, prevalence
✅ Compute Wald confidence intervals
✅ Compute robust bootstrap confidence intervals

📜 Full R Code (Comprehensive)

```r
r

# =====================================================
# Hui-Walter Latent Class Model - FULL EXAMPLE IN R
# =====================================================

# Load required packages
library(tidyverse)
library(boot)

# =====================================================
# STEP 1: Simulate data
# =====================================================

# True parameter values
true_params <- list(
  Se1 = 0.85,
  Sp1 = 0.90,
  Se2 = 0.80,
  Sp2 = 0.95,
  pi1 = 0.30,
  pi2 = 0.60
)

# Function to simulate one population
simulate_population <- function(n, pi, Se1, Sp1, Se2, Sp2) {
  D <- rbinom(n, 1, pi)
  T1 <- ifelse(D == 1, rbinom(n, 1, Se1), rbinom(n, 1, 1 - Sp1))
  T2 <- ifelse(D == 1, rbinom(n, 1, Se2), rbinom(n, 1, 1 - Sp2))
```

```
  data.frame(D, T1, T2)
}

# Simulate populations
set.seed(123)

n1 <- 1000
n2 <- 1000

pop1 <- simulate_population(n1, true_params$pi1, true_params$Se1, true_params$Sp1,
true_params$Se2, true_params$Sp2) %>%
  mutate(pop = 1)
pop2 <- simulate_population(n2, true_params$pi2, true_params$Se1, true_params$Sp1,
true_params$Se2, true_params$Sp2) %>%
  mutate(pop = 2)

# Combine data
data_all <- bind_rows(pop1, pop2)

# Create contingency table
table_data <- data_all %>%
  group_by(pop, T1, T2) %>%
  summarise(count = n()) %>%
  ungroup()

cat("\n--- Contingency table of test outcomes ---\n")
print(table_data)

# ======================================================
# STEP 2: Define log-likelihood for Hui-Walter model
# ======================================================

loglik <- function(params, table_data) {
  Se1 <- params[1]
  Sp1 <- params[2]
  Se2 <- params[3]
  Sp2 <- params[4]
  pi1 <- params[5]
  pi2 <- params[6]

  logL <- 0

  for (k in 1:2) {
    pi_k <- ifelse(k == 1, pi1, pi2)

    sub_table <- table_data %>% filter(pop == k)

    for (i in 0:1) {
      for (j in 0:1) {
        count_ij <- sub_table %>% filter(T1 == i, T2 == j) %>% pull(count)
        if (length(count_ij) == 0) count_ij <- 0

        # Probability of (i,j) pattern
        p_ij <- pi_k *
          (ifelse(i == 1, Se1, 1 - Se1)) *
          (ifelse(j == 1, Se2, 1 - Se2)) +
          (1 - pi_k) *
          (ifelse(i == 1, 1 - Sp1, Sp1)) *
          (ifelse(j == 1, 1 - Sp2, Sp2))

        p_ij <- max(p_ij, 1e-10)  # Avoid log(0)

        logL <- logL + count_ij * log(p_ij)
      }
    }
  }

  return(-logL)  # Negative log-likelihood
}

# ======================================================
# STEP 3: Fit model (maximum likelihood estimation)
# ======================================================

# Initial parameter guesses
```

```r
init_params <- c(0.8, 0.8, 0.8, 0.8, 0.5, 0.5)

# Optimization
fit <- optim(par = init_params,
             fn = loglik,
             table_data = table_data,
             method = "L-BFGS-B",
             lower = rep(0.01, 6),
             upper = rep(0.99, 6),
             hessian = TRUE)

# Extract estimates
est_params <- fit$par
names(est_params) <- c("Se1", "Sp1", "Se2", "Sp2", "pi1", "pi2")

# Print estimates
cat("\n--- Parameter estimates (MLE) ---\n")
print(est_params)

# =====================================================
# STEP 4: Wald confidence intervals (from Hessian)
# =====================================================

# Compute standard errors
se_params <- sqrt(diag(solve(fit$hessian)))

# Wald CIs
wald_ci <- tibble(
  parameter = names(est_params),
  estimate = est_params,
  lower = est_params - 1.96 * se_params,
  upper = est_params + 1.96 * se_params
)

cat("\n--- Wald confidence intervals ---\n")
print(wald_ci)

# =====================================================
# STEP 5: Bootstrap for robust confidence intervals
# =====================================================

# Bootstrap function
bootstrap_fn <- function(data, indices) {
  data_boot <- data[indices, ]

  table_boot <- data_boot %>%
    group_by(pop, T1, T2) %>%
    summarise(count = n()) %>%
    ungroup()

  fit_boot <- optim(par = init_params,
                    fn = loglik,
                    table_data = table_boot,
                    method = "L-BFGS-B",
                    lower = rep(0.01, 6),
                    upper = rep(0.99, 6))

  return(fit_boot$par)
}

# Prepare data for bootstrapping
data_long <- data_all

# Run bootstrap
set.seed(123)
boot_res <- boot(data = data_long, statistic = bootstrap_fn, R = 500)

# Compute bootstrap percentile CIs
boot_ci <- tibble(
  parameter = names(est_params),
  estimate = est_params,
  lower = apply(boot_res$t, 2, quantile, probs = 0.025),
  upper = apply(boot_res$t, 2, quantile, probs = 0.975)
)
```

```
cat("\n--- Bootstrap confidence intervals ---\n")
print(boot_ci)

# =====================================================
# END OF SCRIPT
# =====================================================
```

🚀 Summary

This **comprehensive script**:

✅ Simulates data under **Hui-Walter assumptions**
✅ Defines and fits **log-likelihood** for latent class model
✅ Estimates:

- Sensitivity of both tests
- Specificity of both tests
- Prevalence in both populations

✅ Provides:

- **Wald confidence intervals** (based on Hessian / Fisher information)
- **Bootstrap confidence intervals** (robust, non-parametric)

🔎 How to use

1️⃣ Copy full script into RStudio or an R script (.R) file
2️⃣ Run all
3️⃣ You will see:

- Joint test outcome table
- MLE estimates
- Wald CIs
- Bootstrap CIs

🚩 Possible Extensions

If you want, I can also prepare:
✅ Hui-Walter with **3 tests**
✅ Hui-Walter with **>2 populations**
✅ **Bayesian version** (Stan or JAGS)
✅ Add **local dependence** (for correlated tests)
✅ Wrap everything in a **clean R function** (easy to reuse)

Would you like me to generate any of these next?
Just tell me — I'll be happy to customize the code for your exact needs! 🚀
